# The impact of pain on quality of life in patients with osteoarthritis: a cross-sectional study from Palestine

**DOI:** 10.1186/s12891-022-05207-x

**Published:** 2022-03-14

**Authors:** Mojahed Shalhoub, Mohammad Anaya, Soud Deek, Anwar H. Zaben, Mazen A. Abdalla, Mohammad M. Jaber, Amer A. Koni, Sa’ed H. Zyoud

**Affiliations:** 1 Department of Medicine, College of Medicine and Health Sciences, An-Najah National University, Nablus, 44839 Palestine; 2 Department of Orthopedic Surgery, An-Najah National University Hospital, Nablus, 44839 Palestine; 3 Division of Clinical Pharmacy, Department of Hematology and Oncology, An-Najah National University Hospital, Nablus, 44839 Palestine; 4 Department of Clinical and Community Pharmacy, College of Medicine and Health Sciences, An-Najah National University, Nablus, 44839 Palestine; 5 Poison Control and Drug Information Center (PCDIC), College of Medicine and Health Sciences, An-Najah National University, Nablus, 44839 Palestine; 6 Clinical Research Center, An-Najah National University Hospital, Nablus, 44839 Palestine

**Keywords:** Osteoarthritis, Quality of life, Pain, Brief pain inventory, EQ-5D, EQ-VAS, Palestine

## Abstract

**Background:**

Osteoarthritis is one of the most common musculoskeletal problems. Pain is the most common complaint and the most significant cause of decreased health-related quality of life (HRQOL) among osteoarthritic patients. The objectives of this study were to assess the impact of pain on quality of life among patients with osteoarthritis and to assess the association of sociodemographic and clinical factors with HRQOL.

**Methods:**

Using a cross-sectional study design, we collected data from osteoarthritis patients in orthopedic outpatient clinics from four hospitals in the Palestine-West bank between November 2020 and March 2021. We used the Brief Pain Inventory (BPI) scale to assess pain and the Quality of Life scale five dimensions (EQ-5D) with the visual analog scale of the European Quality of Life (EQ-VAS) to assess HRQOL.

**Results:**

In our study, 196 patients composed the final sample, with an average of 60.12 ± 13.63 years. The medians for the EQ-5D score and EQ-VAS score were 0.72 (0.508–0.796) and 70 (55–85), respectively. The pain severity score was found to have a significant negative association with both the EQ-5D and EQ-VAS scores with r of − 0.620, *p* <  0.001, and − 0.554, *p* <  0.001, respectively. Similar associations were found between pain interference score and both EQ-5D (*r* = − 0.822, *p* <  0.001) and EQ-VAS scores (*r* = − 0.609, *p* <  0.001). Multiple regression analysis showed that participants with higher educational level (*p* = 0.028), less diseased joints (*p* = 0.01), shorter duration of disease (*p* = 0.04), and lesser pain severity and interference scores (both with *p* < 0.001) had significantly higher HRQOL scores.

**Conclusions:**

We found that many variables have a significant negative impact on HRQOL among patients with osteoarthritis. Our finding provides a well-founded database to use by clinicians and healthcare professionals who work with patients with osteoarthritis, as well as educational and academic institutions.

## Background

Osteoarthritis (OA) is an age-related disease characterized pathologically by areas of focal damage and loss of articular cartilage in synovial joints [1], is one of the most frequent chronic diseases that can lead to loss of quality of life and increased prevalence and incidence due to increased life expectancy [1]. Worldwide, osteoarthritis affects approximately 7% of the population [2]. The primary clinical symptom of osteoarthritis is pain, which can be intermittent or constant [3]. Pain is the symptom that forces patients to seek medical advice and contributes the most to functional limitations and reduced quality of life [3]. Specifically, the impact of OA on the quality of life was found to be significantly associated with the sites of pain and sex [4].

However, pain is not the only symptom of osteoarthritis. Patients may also suffer from joint stiffness, especially in the morning [5], and joint cracking during movement [6]. Osteoarthritis can be diagnosed by taking a complete comprehensive history and physical examination [7]. The diagnosis may or may not require radiographic findings, considering that some patients may initially be asymptomatic [7].

Osteoarthritis is classified into two main types based on previous abnormalities in the affected joint. The first type is primary osteoarthritis, which occurs in joints without a previous abnormality and an inciting trauma or agent. The second type is secondary osteoarthritis, which is more common than the primary type. It is usually due to a previous joint abnormality, such as trauma, rheumatoid arthritis, avascular necrosis, hemoglobinopathy, Paget disease, Ehlers-Danlos syndrome, or Marfan syndrome [8, 9].

There are many risk factors for osteoarthritis, some of which are modifiable, and some are nonmodifiable. The most important modifiable factors are obesity, occupational status, comorbidities, and physical activity that can be managed to improve joint function. Nonmodifiable risk factors include age and genetic or hereditary mutations that increase the susceptibility to osteoarthritis [10, 11].

In Europe, it was reported that a large percentage of OA patients (59.6%) complained of moderate to severe pain and had a significant impact on several aspects of health [12]. It was also documented that patients with moderate to severe pain due to OA had a high impact on the quality of life, even using medications [12]. In addition, the OA population had lower social relationships, psychological well-being, and independent living than individuals without OA [13]. That necessitates appropriate intervention to enhance their HRQOL [13]. Due to the impact of osteoarthritis on quality of life, many therapies are used to improve symptoms in these patients, although no treatment delays or prevents osteoarthritis or provides long-term relief of symptoms [14]. In general, OA treatment options depend on the severity and duration of patient symptoms. They include non-pharmacological (i.e., physical therapy), pharmacological (i.e., acetaminophen and non-steroidal anti-inflammatory drugs), complementary (i.e., yoga and acupuncture), and surgical options (i.e., joint replacement) [7];.

According to an extensive review, OA has a significant effect on the quality of life [15]. Knowledge about HRQOL is vital for building up a specific treatment plane tailoring each patient, considering the predictors that most affect the HRQOL [15]. Notably, these factors are varied among different countries and regions, and therefore, they should be studied in different cohorts of patients. In the literature, there is an information gap about Palestinian patients with OA. In addition, little data are available about the impact of sociodemographic and clinical characteristics together on OA patients’ quality of life [16]. Therefore, in this study, we collected information from OA patients about their pain using the Brief Pain Inventory (BPI) and assess its impact on daily activity by the five-level EuroQol five-dimensional instrument (EQ-5D-5L). The results will help provide information on the effectiveness of pain management and the impact of different patients’ characteristics to improve quality of life and encourage the incorporation of psychosocial assessment into management.

## Methods

### Study design and setting

We conducted a cross-sectional study over 6 months, between November 2020 and March 2021, in the orthopedic outpatient clinics of four main hospitals; three of them were governmental and included Rafidia Governmental Hospital, Qalqilia Governmental Hospital, and Tulkarem Governmental Hospital, and the fourth was an academic hospital, which was An-Najah National University Hospital (NNUH).

### Study population and sample size

To carry out our study, we chose four hospitals in northern Palestine. We calculated the size of our sample using the Raosoft Inc. sample size calculator. (http://www.raosoft.com/samplesize.html). Convenient sampling was used to select 196 patients, distributed as the following: 100 patients were recruited from the Rafidia Governmental Hospital, 35 patients from the Qalqilia Governmental Hospital, 20 patients from the Tulkarem Governmental Hospital, and 41 patients from the NNUH. We relied on the availability in these hospitals to decide the number of participants from each hospital, such as the fraction of patients in each hospital was somewhat representative of the percentage of patients who counseled the outpatient clinic of the whole number of patients counseled in all selected hospitals.

### Inclusion and exclusion criteria

To be included in this study, the patient must be over 18 years, have had osteoarthritis for 6 months or more, and be on any pain medication for osteoarthritis. In addition, patients who refused to give their consent or had mental retardation, psychiatric disorders, or communication problems and those who had significant visual, vestibular, neurological, peripheral, or sensory disorders were excluded from our study.

### Data questionnaire

The selected patients were assessed using a 4-section questionnaire. The first part inquired about the social and demographic characteristics of the participants, including age, sex, marital status, educational level, residency, occupation, alcohol, and smoking status. We classified smoking status into smokers and nonsmokers for analysis, and age was subclassified into two groups: less than 40 years and 40 years or more.

The second section included questions and items about clinical data related to osteoarthritis, including body mass index (BMI), duration of osteoarthritis, number of affected joints, any previous trauma to the affected joint, pain medications used, and number of chronic diseases of any type. For analysis purposes, the BMI was categorized into normal weight (18.5–24.9), overweight (25–29.9), and obese (30 or more), and pain medications were classified as acetaminophen, NSAIDs, or both.

In the third section, we included a tool for pain measurement, the Arabic version of the Brief Pain Inventory (BPI), which is an accepted and widely used tool for the assessment of pain in terms of intensity (sensory dimension) and interference with the patient’s life (reactive dimension) [17]. We completed the application and received permission for using the validated Arabic version of the short form BPI of the MD Anderson Cancer Center [17–19]. In Lebanese research, it was found that the Arabic version of that scale has shown validity, reliability, and cultural sensitivity when used in patients with Lebanese oncology patients [20]. Regarding the severity of pain, the following severity domains were evaluated: the degree of the most severe pain which was felt during the last 24 h, the degree of the mildest pain that was felt during the last 24 h, the average degree of pain felt during the last 24 h, and the degree of pain felt at the time of assessment. Each of these aspects was scored from 0 to 10, and then we calculated the pain severity score and classified the scores as mild, moderate, and severe. Specifically, the total score ranges between 0 and 40, which was converted into a 10-point scale. A score of ≤4 was considered mild, > 4–6 was moderate, and > 6 was severe. For the interference of pain with daily functions, the following items were assessed: the effect of pain on walking ability, its interference with the general mood and its effect on working ability, general activity, quality of sleep, social relationships, and enjoyment of life. Each of these aspects was scored from 0 to 10, and then we calculated the Pain Interference scale and classified the results into low and high interference. Patients with a total 10-point score of ≤5 were considered ‘low interference’, while those who have a score of > 5 were marked as ‘high interference’. It also contained other questions that address specific sites of pain, types of pain management methods used, and the degree of pain relief with these methods.

For the last part of the questionnaire, we used an assessment tool for health-related quality of life (HRQOL), which is the European Quality of Life Scale 5 dimensions 5 levels (EQ-5D-5L). The EQ-5D-5L is a widely used tool to evaluate the overall quality of life [21, 22], and has good reliability and validity in measuring HRQOL in Palestine [23–29]. In its first section, it contains five major parts that cover the following aspects of quality of life: mobility or ability to walk, self-care activities such as self-washing or self-dressing, usual daily activities such as working, studying, or housework, pain and/or discomfort, and anxiety and/or depression [21]. Then, we used the results of this section to calculate Crosswalk Index values. Then, we used the Crosswalk Index Calculator of the EQ-5D-5L [30] based on the scoring algorithm of the USA general population. In the second part, the EQ-5D-5L contains the EQ-VAS scale, a 0 to 100 visual analogue scale that allows the participants to estimate their HRQOL, with 0 being the worst imaginable and 100 denoting the full health status. The Euro-QOL Research Foundation permitted us to use the Arabic version of the EQ-5D in this study (registered ID: 41391).

To evaluate its understandability and simplicity and how much time is needed to complete it, we tested the questionnaire in a pilot study on ten patients who were not included in the final study.

### Ethical approval

The *Institutional Review Board (IRB) of An-Najah National University* (# 21 October 2020) approved the study. Permission was obtained from selected hospitals to allow researchers to interview their patients.

### Data analysis

We analyze the data in our study using the IBM-SPSS version 26. Frequencies and percentages were used to present the qualitative variables, while means and standard deviations or medians and interquartile ranges were used to describe the continuous variables. We used the Kolmogorov-Smirnov test to evaluate the normality of continuous variables. Then, Mann-Whitney and Kruskal-Wallis tests were applied to assess the significance of correlations between patient characteristics and their scores on the scales used in this study. We considered *p*-values of < 0.05 to be significant. Furthermore, we used multiple regression analysis to determine the specific variables that were independently associated with quality of life. All variables that are significantly correlated with quality of life, including sociodemographic, clinical, as well as pain severity and interference, in the bivariate analysis were entered into the regression model. We also checked the internal consistency of the Pain Severity Score, Pain Interference Score, and HRQOL scale using the Cronbach alpha test.

## Results

### Demographic and clinical characteristics

Two hundred twenty-one participants were invited to participate in this study; 196 of them agreed to participate, resulting in a response rate of 89%. Table 1 details the demographic and clinical variables of the study sample. We found that the mean age in years was 60.2 ± 13.63, with 94.4% of them at 40 years or more. The preponderance of the participants (61.2%) was female, and 66.3% of all participants were married. Approximately half of the participants (46.4%) lived in cities, 64.8% did not have an occupation, and 55.6% were considered obese.

**Table 1:** Sociodemographic and clinical characteristics of the study population

Variable	Frequency (%) ***N*** = 196
**Gender**	
Male	76 (38.8)
Female	120 (61.2)
**Age category (years)**	
< 40	11 (5.6)
≥40	185 (94.4)
**Marital status**	
(Single, Divorced, Widowed)	66 (33.7)
Married	130 (66.3)
**Residency**	
Village	84 (42.9)
Camp	21 (10.7)
City	91 (46.4)
**Educational level**	
No formal education	40 (20.4)
Primary	31 (15.8)
Preparatory	45 (23.0)
Secondary	39 (19.9)
University	41 (20.9)
**Smoking status**	
Smoker	62 (31.6)
Non-smoker	134 (68.4)
**Occupation**	
Not working	127 (64.8)
Working	69 (35.2)
**BMI**	
Normal weight	21 (10.7)
Overweight	66 (33.7)
Obese	109 (55.6)
**Trauma**	
Yes	64 (32.7)
No	132 (67.3)
**Pain medications**	
Acetaminophen only	51 (26.0)
NSAIDs only	74 (37.8)
Acetaminophen & NSAIDs	71 (36.2)
**Number of affected joints**	
One joint	83 (42.3)
Two joints	87 (44.4)
Three joints or more	26 (13.3)
**Duration of the disease**	
Less than five years	76 (38.8)
5–10 years	81 (41.3)
More than ten years	39 (19.9)
**Number of comorbidities**	
0	51 (26.0)
1	67 (34.2)
2	44 (22.4)
3 or more	34 (17.3)
**Pain severity**	
Mild	100 (51.0)
Moderate	59 (30.1)
Sever	37 (18.9)
**Pain interference**	
Low	131 (66.8)
High	65 (33.2)
**Percentage of relief by medications**	
30% or less	15 (7.7)
40–60%	56 (28.6)
70–100%	125 (63.8)

*Abbreviations*: *BMI* Body mass index

Regarding the characteristics related to osteoarthritis, 41.3% had the disease for 5 to 10 years, and 38.8% had the disease for less than 5 years. Of all 196 subjects, 67.3% reported no history of trauma to the affected joint, 44.4% had two affected joints, and 42.3% had only one affected joint. Approximately half (51%) of the participants had mild pain, while the majority of them (66.8%) had a low pain interference score. Table 1 shows the detailed frequencies and percentages of demographic and clinical characteristics among the participants. The two most commonly reported pain sites were the right (67.3%) and left (62.2%) knees. Table 2 shows the frequencies and percentages of various reported pain sites.

**Table 2:** Site of the affected joint

Affected joint	Frequency (%) ***N*** = 196
Right knee	132 (67.3)
Left knee	122 (62.2)
Right ankle	11 (5.6)
Left ankle	15 (7.7)
Right hip	4 (2.0)
Left hip	4 (2.0)
Right wrist	5 (2.6)
Left wrist	4 (2.0)
Right elbow	11 (5.6)
Left elbow	10 (5.1)
Right shoulder	6 (3.1)
Left shoulder	4 (2.0)
Spine	7 (3.6)

### Pain severity and interference scores

In our study, the mean pain severity score was 4.23 ± 2.09 with a median of 4 (2.75–5.5), while the mean pain interference score was 3.94 ± 2.33 with a median of 3.85 (2.0–5.68). Furthermore, the Cronbach alpha of the pain severity score was 0.906, and that of the pain interference score was 0.881, which indicates that these scores have good internal reliability.

### EQ-5D and EQ-VAS scores

We found that the median of the EQ-5D score of our sample was 0.72 with the interquartile range (0.51–0.80) and it is mean was 0.65 ± 0.19. Cronbach’s alpha was used to measure internal consistency, and our result was 0.867, indicating good internal reliability. Regarding the EQ-VAS score, the median score of our study was 70.00 (55.00–85.00), while its mean score was 70.43 ± 19.14.

**Fig. 1:**
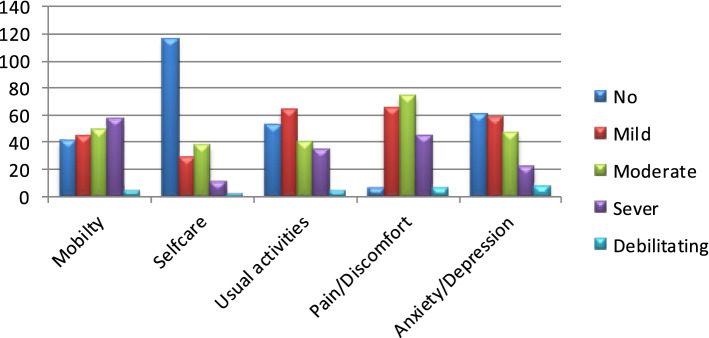
Distribution of different quality of life indices in different domains of the five dimensions European Quality of Life scale (EQ-5D)

The worst health status in all dimensions of the EQ-5D was reported as follows: mobility 2%, self-care 1%, usual activity 2%, pain and or restlessness 3.1%, and anxiety and/or depression 4.1%. Furthermore, the worst condition in any dimension was reported by 7.7% of the sample (Fig. 1).

### EQ-5D score’s univariate and multiple linear regression analysis

Table 3 summarizes the univariate analysis of the EQ-5D score of our sample by their characteristics. It shows that the EQ-5D score was significantly associated (*p* < 0.05) with age, educational level, occupation, previous trauma to the joint, number of joints affected, number of comorbidities, duration of the disease, and severity and interference pain scores of the patients. In addition, in our study, a significant negative association was found between the EQ-5D score and both the pain interference score (r of − 0.822, *p* < 0.001) and the pain severity score (*r* = − 0.620, *p* < 0.001).

**Table 3:** The five dimensions of the European Quality of Life scale (EQ-5D) of the participants by demographic and clinical characteristics

Variable	Median(Q1-Q3)	***P*** value^**1**^
**Gender**		
Male	0.74 (0.54–0.80)	0.337^2^
Female	0.70 (0.49–0.79)	
**Age category (years)**		
< 40	0.80 (0.79–0.81)	**0.001**^**2**^
≥40	0.75 (0.50–0.79)	
**Marital status**		
(Single, Divorced, Widowed)	0.73 (0.59–0.80)	0.561^2^
Married	0.72 (0.50–0.80)	
**Residency**		
Village	0.71 (0.51–0.81)	0.478^3^
Camp	0.64 (0.49–0.76)	
City	0.73 (0.54–0.80)	
**Educational level**		
No formal education	0.64 (0.44–0.79)	**0.047**^**3**^
Primary	0.71 (0.47–0.78)	
Preparatory	0.66 (0.47–0.78)	
Secondary	0.73 (0.62–0.81)	
University	0.76 (0.67–0.80)	
**Smoking status**		
Smoker	0.73 (0.53–0.80)	0.792^2^
Non-smoker	0.72 (0.50–0.80)	
**Occupation**		
Not working	0.69 (0.47–0.79)	**0.011**^**2**^
Working	0.74 (0.64–0.80)	
**BMI**		
Normal weight	0.74 (0.55–0.80)	0.051^3^
Overweight	0.74 (0.56–0.82)	
Obese	0.66 (0.49–0.78)	
**Trauma**		
Yes	0.79 (0.72–0.81)	**< 0.001**^**2**^
No	0.65 (0.46–0.76)	
**Pain medications**		
Acetaminophen only	0.76 (0.63–0.82)	**0.002**^**3**^
NSAIDs only	0.73 (0.54–0.79)	
Acetaminophen & NSAIDs	0.64 (0.43–0.76)	
**Number of affected joints**		
One joint	0.76 (0.70–0.82)	**< 0.001**^**3**^
Two joints	0.65 (0.48–0.76)	
Three joints or more	0.63 (0.37–0.74)	
**Duration of the disease**		
Less than five years	0.74 (0.62–0.81)	**0.006**^**3**^
5–10 years	0.72 (0.49–0.83)	
More than ten years	0.62 (0.44–0.74)	
**Number of comorbidities**		
0	0.76 (0.69–0.83)	**< 0.001**^**3**^
1	0.73 (0.53–0.79)	
2	0.67 (0.47–0.78)	
3 or more	0.61 (0.37–0.78)	
**Pain severity**		
Mild	0.78 (0.72–0.81)	**< 0.001**^**3**^
Moderate	0.63 (0.47–0.78)	
Sever	0.46 (0.34–0.54)	
**Pain interference**		
Low	0.78 (0.62–0.82)	**< 0.001**^**2**^
High	0.47 (0.35–0.61)	
**Percentage of relief by medications**		
30% or less	0.50 (0.37–0.64)	**< 0.001**^**3**^
40–60%	0.51 (0.37–0.71)	
70–100%	0.76 (0.66–0.81)	

Abbreviation: BMI, Body mass index

^1^Bold values denote statistical significance at the level of *p* < 0.05 level of the association

^2^Mann-Whitney test

^3^Kruskal-Wallis test

Table 4 reveals the multiple linear regression analysis of the association between the characteristics and the EQ-5D score. The results showed that the participants with higher educational level (*p* = 0.028), less diseased joints (*p* = 0.01), shorter duration of disease (*p* = 0.04) and lesser scores of pain severity (*p* < 0.001) and pain interference (*p* < 0.001) had significantly higher HRQOL scores.

**Table 4:** Multiple regression analysis of the characteristics with their quality of life (EQ-5D-Score)

Variables	Unstandardized coefficients		Standardized coefficients	T	***P*** value^**1**^	95.0% confidence interval for B		Collinearity statistics
	B	Std. error	Beta			Lower bound	Upper bound	VIF
**Constant**	0.915	0.090		10.206	0.000	0.738	1.092	
**Age**	0.034	0.025	0.070	1.377	0.170	−0.015	0.083	1.342
**Educational level**	0.015	0.007	0.112	2.217	**0.028**	0.002	0.029	1.336
**Occupation**	0.002	0.007	0.011	0.234	0.815	−0.012	0.015	1.232
**Trauma**	−0.001	0.022	−0.003	− 0.051	0.959	− 0.044	0.041	1.436
**Pain medications**	−0.017	0.012	−0.068	−1.452	0.148	−0.040	0.006	1.146
**Number of affected joints**	−0.035	0.013	−0.123	−2.592	**0.010**	−0.061	−0.008	1.183
**Duration of disease**	−0.027	0.013	−0.102	−2.070	**0.040**	−0.052	−0.001	1.272
**Number of comorbidities**	−0.007	0.010	−0.036	−0.703	0.483	−0.026	0.012	1.393
**Pain severity**	−0.061	0.015	−.245	−4.110	**< 0.001**	−0.091	−0.032	1.872
**Pain interference**	−0.179	0.023	−0.437	−7.729	**< 0.001**	−0.225	−0.133	1.675
**Percentage of relief by drugs**	0.046	0.016	0.150	2.829	**0.005**	0.014	0.078	1.477

^1^Bold values denote statistical significance at the level of *p* < 0.05 level

### EQ-VAS score univariate and multiple linear regression analysis

Table 5 summarizes the univariate analysis of the EQ-VAS score of our sample by their characteristics. The EQ-VAS score showed a significant correlation with gender, age, educational level, occupation, trauma, pain medications, number of affected joints, duration of the disease, number of comorbidities, pain interference, and percentage of pain relief by medications. The EQ-VAS score was also found to have a significant negative association with both the pain severity score (*r* = − 0.554, *p* < 0.001) and the pain interference score (*r* = − 0.609, *p* < 0.001), while it was positively correlated with the EQ-5D score (r of 0.618, *p* < 0.001).

**Table 5:** Visual analogue scores of European Quality of Life of participants by demographic and clinical characteristics

Variable	Median(Q1-Q3)	***P*** value^**1**^
**Gender**		
Male	75 (61.25–90)	**0.048**^**2**^
Female	70 (55–80)	
**Age category (years)**		
< 40	95 (90–100)	**< 0.001**^**2**^
≥40	70 (55–80)	
**Marital status**		
(Single, Divorced, Widowed)	72.5 (50–90)	0.674^2^
Married	70 (60–80)	
**Residency**		
Village	70 (50–80)	0.143^3^
Camp	75 (57.5–82.5)	
City	75 (60–90)	
**Educational level**		
No formal education	70 (50–83.75)	**< 0.001**^**3**^
Primary	70 (50–85)	
Preparatory	70 (50–75)	
Secondary	70 (70–80)	
University	90 (70–95)	
**Smoking status**		
Smoker	75 (55–95)	0.098^2^
Non-smoker	70 (58.75–80)	
**Occupation**		
Not working	70 (50–80)	**< 0.001**^**2**^
Working	80 (70–95)	
**BMI**		
Normal weight	80 (70–95)	0.137^3^
Overweight	70 (50–85)	
Obese	70 (57.5–80)	
**Trauma**		
Yes	85 (75–95)	**< 0.001**^**2**^
No	70 (50–75)	
**Pain medications**		
Acetaminophen only	80 (70–95)	**< 0.001**^**3**^
NSAIDs only	75 (60–86.25)	
Acetaminophen & NSAIDs	70 (50–75)	
**Number of affected joints**		
One joint	80 (70–95)	**< 0.001**^**3**^
Two joints	70 (50–80)	
Three joints or more	60 (38.75–75)	
**Duration of the disease**		
Less than five years	80 (70–95)	**0.001**^**3**^
5–10 years	70 (52.5–80)	
More than ten years	70 (50–80)	
**Number of comorbidities**		
0	85 (70–95)	**< 0.001**^**3**^
1	70 (60–80)	
2	70 (50–83.75)	
3 or more	55 (35–70)	
**Pain severity**		
Mild	80 (70–95)	**< 0.001**^**3**^
Moderate	70 (50–75)	
Sever	60 (45–70)	
**Pain interference**		
Low	80 (70–95)	**< 0.001**^**2**^
High	55 (40–70)	
**Percentage of relief by medication**		
30% or less	70 (60–80)	**< 0.001**^**3**^
40–60%	55 (45–70)	
70–100%	80 (70–90)	

*Abbreviation*: *BMI* Body mass index

^1^Bold values denote statistical significance at the level of *p* < 0.05 level

^2^Mann-Whitney test

^3^Kruskal-Wallis test

The results of the multiple linear regression analysis of the correlation with the EQ-VAS score are summarized in Table 6, which showed that participants with previous trauma (*p* = 0.007), lower number of comorbidities (*p* = 0.007), lower number of affected joints (*p* = 0.002), lower severity of pain severity (*p* = 0.008) and pain inference (*p* = 0.001) had significantly higher VAS scores.

**Table 6:** Multiple regression analysis of the association of participant characteristics with their visual analog scores

Variables	Unstandardized coefficients	Standardized coefficients		T	***P*** value^**1**^	95.0% confidence interval for B		Collinearity statistics
	B	Std. error	Beta			Lower bound	Upper bound	VIF
**Constant**	107.316	11.140		9.633	.000	85.335	129.296	
**Gender**	1.846	2.268	0.047	0.814	0.417	−2.629	6.321	1.357
**Age**	−0.820	2.781	−0.017	− 0.295	0.768	−6.306	4.667	1.343
**Educational level**	0.837	0.782	0.062	1.070	0.286	−0.706	2.380	1.364
**Occupation**	1.502	0.855	0.106	1.757	0.081	−0.184	3.189	1.470
**Trauma**	−6.686	2.429	−0.164	−2.753	**0.007**	−11.478	−1.894	1.441
**Pain medications**	−2.243	1.304	−0.092	−1.721	0.087	−4.815	0.329	1.155
**Number of affected joints**	−4.737	1.506	−0.170	−3.146	**0.002**	−7.708	−1.766	1.188
**Duration of disease**	−1.603	1.478	−0.062	−1.085	0.279	−4.519	1.312	1.337
**Number of comorbidities**	−2.922	1.078	−0.159	−2.710	**0.007**	−5.050	−0.795	1.397
**Pain severity**	−4.514	1.687	−0.182	−2.676	**0.008**	−7.841	−1.186	1.882
**Pain interference**	−9.264	2.614	−0.228	−3.543	**0.001**	−14.422	−4.106	1.683
**Percentage of relief by drugs**	2.333	1.825	0.077	1.279	0.203	−1.267	5.934	1.477

^1^Bold values denote statistical significance at the level of *p* < 0.05 level

## Discussion

This cross-sectional study conducted a thorough analysis of HRQOL in OA patients on pain management in four hospitals in the north of the Palestinian West Bank. We quantified HRQOL using the EQ-5D-5L scale and its VAS component, in addition to the Brief Pain Inventory (BPI) scale to assess the severity of pain and its interference in our sample. Different sociodemographic and clinical factors are correlated with HRQOL. We found that HRQOL was lower in patients with demographic characteristics, such as older age, lower educational level, and unemployment.

Patients with OA suffer from various clinical manifestations, such as pain, which have an impact on HRQOL [3]. Therefore, several studies have used the EQ-5D scale to measure and evaluate HRQOL in patients with OA [31–37]. As this scale can detect changes in HRQOL, i.e., deterioration and improvements, it can determine the differences and effects of some interventions on HRQOL among OA patients.

The mean of the EQ-5D score among participants was 0.65 ± 0.19. This is nearly the same as what was found in some previous studies, which reported scores of 0.66 ± 0.24 and 0.67 ± 0.3 in Sweden [35, 36] and 0.68 ± 0.23 in China [37]. However, the EQ-5D score in our study was higher than the mean score of three similar studies in Spain [31, 33, 34], which could be explained by differences in sociodemographic and clinical factors that affect the HRQOL, such as age, education level, and employment.

Multiple factors were associated with HRQOL in Palestinian patients with OA, which confirmed the findings of the previous studies [29, 34, 38–43]. In addition, the correlation between aging and HRQOL that our study showed is comparable to that previously found in studies from Spain [34, 38, 39] and Iran [40]. This could be due to the effect of OA on physical activity, along with the social withdrawal associated with advanced age, which will negatively affect their HRQOL [41].

We have also found that educational level and occupation were significantly associated with HRQOL; with lower HRQOL in uneducated and unemployed patients. This is quite similar to a previous study in Brazil [41]. This could be related, in part, to the negative effect of inactivity on HRQOL [44] as these patients are vulnerable to having less physical activity, which also results in higher rates of stress and depression [45, 46]. This unemployment factor seems to be a cofounder as unemployed patients may be at increased risk of having lower HRQOL, or patients with lower HRQOL may be more likely to be unemployed. In addition, elderly patients are more likely to have lower HRQOL, but at the same time, they are more likely to be unemployed as well.

We found a significant association between a higher number of comorbidities and a lower HRQOL, a finding comparable to the results of a previous study [42]. Although the number of comorbidities affects HRQOL, the type of comorbidities may give a deep understanding of this association. That is similarly applied to the site of the affected joint. Therefore, studies of the correlations between each component of the comorbidities or the site of the affected joint with the HRQOL are recommended. Furthermore, higher pain severity scores were significantly associated with lower HRQOL, a finding similar to results from previous studies [29, 43].

Regarding the EQ-VAS score, our study showed a mean of 70.43 ± 19.14, higher than similar studies in Spain [31, 34]. Furthermore, the EQ-5D score was positively associated with EQ-VAS, which indicates that it may be more accurate to use both scores together for HRQOL assessment than each one alone. Finally, HRQOL was found to have a significant negative association with both pain severity and interference scores. Similar results have been reported in a previous publication, emphasizing the importance of pain management and not ignoring pain complaints, especially for those with inadequate pain relief [47].

### Strengths and limitations

The strengths of our study include the fact that it is conducted in multiple hospitals in the West Bank in Palestine. This study was the first study in Palestine to evaluate HRQOL among OA patients and examine its association with pain control, in addition to various clinical and sociodemographic variables. Moreover, the information was gathered through face-to-face interviews, making our data complete and reliable. However, since data collectors performed face-to-face interviews with patients, this might have negative outcomes because it could influence participants’ answers to the main questions. Moreover, this study has some limitations that include using convenience sampling techniques that may reduce the generalizability to our study population. We also use a cross-sectional design that does not clarify the cause-effect relationship. Additionally, as our study was carried out in the outpatient clinics of hospitals, that might overestimate the severity of the pain. Notably, only one specific system is applied, which may be not convincing and reliable as there are several score systems of assessments of function and quality of life. Furthermore, specific clinical data, such as classification and severity of OA were not determined and some sociodemographic variables, such as educational level and occupation are a potential risk of bias since it is a kind of multi-factor issue. In this study, we did not conduct a subgroup analysis or test the correlations between the type of comorbidities or the site of the affected joint with the HRQOL. These analyses may give a greater understanding of OA patients’ quality of life. Finally, due to the COVID-19 epidemic, the number of patients available for participation in this study was somewhat limited, which could further reduce the generalizability of the results.

## Conclusions

We found that many variables can remarkably lower the HRQOL in patients with OA. Patients who were older, unemployed, with a lower educational level, a higher number of affected joints, a longer duration of osteoarthritis, and those with multiple comorbidities were at increased risk of having lower HRQOL. The results of our study provide a well-founded database that helps clinicians and healthcare professionals who work with and consult OA patients to achieve higher degrees of pain relief, and therefore, higher quality of life. In addition, it can be useful for educational and academic institutions. Healthcare professionals should pay special attention to the HRQOL in patients with OA, especially those who are risky for worse HRQOL. It is of great importance to advocate standard recommendations and guidelines for the care, treatment, and follow-up of these patients, including pain management as a cornerstone for improving quality of life. However, there is a need for further work on a larger case series. Therefore, we recommend further studies on OA among Palestinians because it is a neglected health issue in previous national investigations.

## Data Availability

The datasets generated and/or analyzed during the current study are not publicly available due to limitations of ethical approval involving the patient data and anonymity but are available from the corresponding author on reasonable request. This manuscript forms part of a Doctor of Medicine graduation project submitted to at An-Najah National University and the abstract was published as part of self-archiving in institutional repositories (i.e., university repository).

## Abbreviations

OA: Osteoarthritis
BMI: Body mass index
NNUH: An-Najah National University Hospital
BPI: Brief Pain Inventory
EQ-5D: European Quality of Life Scale 5 dimensions
HRQOL: Health-related quality of life
EQ-VAS: European Quality of Life visual analogue scale
EQ-5D-5L: European Quality of Life Scale 5 dimensions 5 levels
IRB: Institutional Review Board
SPSS: Statistical Package for the Social Sciences
